# Monoterpenyl Glycosyltransferases Differentially Contribute to Production of Monoterpenyl Glycosides in Two Aromatic *Vitis vinifera* Varieties

**DOI:** 10.3389/fpls.2017.01226

**Published:** 2017-07-13

**Authors:** Xiang-Yi Li, Ya-Qin Wen, Nan Meng, Xu Qian, Qiu-Hong Pan

**Affiliations:** ^1^Center for Viticulture and Enology, College of Food Science and Nutritional Engineering, China Agricultural University Beijing, China; ^2^Key Laboratory of Viticulture and Enology, Ministry of Agriculture Beijing, China; ^3^Institute of Apiculture Research, Chinese Academy of Agricultural Sciences Beijing, China

**Keywords:** monoterpenyl glycoside, monoterpenyl glycosyltransferase, single nucleotide polymorphism, Vitis vinifera variety

## Abstract

**HIGHLIGHTS**

- A similar trend on accumulation of glycosidically bound monoterpenes was observed in both varieties
- Two *VvGT7* alleles mutations occurred at key sites in Muscat blanc à Petit
- *VvGT14* exerted a major role in production of monoterpenyl glycosides in both varieties

Terpenoids are the major aroma components and generally exist as both free and glycosidically-bound forms, of which nonvolatile glycosides account for a large fraction in grape berries. Our previous study has indicated that differential accumulation of monoterpenyl glycosides in *Vitis vinifera* “Muscat blanc à Petit” between two regions is closely correlated to monoterpenyl glucosyltransferase (*VvGT14*, XM_002285734.2) transcript abundance. However, it has not been determined yet whether this correlation also exists in other *Vitis vinifera* varieties. This study investigated the evolution of free and glycosidically bound monoterpenes in two *Vitis vinifera* variety “Muscat blanc à Petit” and “Gewurztraminer” under two vintages, and further assessed the relation between the accumulation of bound monoterpenes and two monoterpenyl glycosyltransferase transcript levels. Results showed that free monoterpenes exhibited three evolution patterns in both varieties during berry development of two vintages, whereas glycosidically bound monoterpenes showed a concentration elevation with berry maturation. The *Cis*-rose oxide and geraniol were major components contributing to the aroma odors of “Gewürztraminer” grapes while linalool was major aroma contributor to the “Muscat blanc à Petit grain” grapes. The accumulation of glycosidically bound monoterpenes in both varieties was accompanied with the high expression of *VvGT7* (XM_002276510.2) and *VvGT14*. Only one allele of *VvGT7* was found in the variety “Gewürztraminer” and no mutation was observed in its enzyme active sites. *XB-VvGT7-4* and *XB-VvGT7-5* were two alleles of *VvGT7* detected in “Muscat blanc à Petit grain.” The mutation on its enzyme active site inhibited the activity of *XB-VvGT7-4*, whereas *VvGT7-5* exhibited an alteration on enzyme activity due to the insertion mutation at the position 443. Only one *VvGT14* allele was found in both varieties, and the *VvGT14* allele in both varieties showed the similarity on amino acid sequence. No mutation occurred in active sites of *VvGT14* allele. These indicated that *VvGT7* and *VvGT14* differentially contributed to the production of monoterpenyl glycosides in these *Vitis Vinifera* varieties.

## Introduction

Terpenes are the critical volatile compounds that contribute Muscat-type grape and wine with the floral and fruity aroma (Strauss et al., 1986; Robinson et al., 2014). It has been reported that more than 50 terpenes have been identified with monoterpenes as the major terpenes (Strauss et al., 1986; Martin et al., 2012). Generally, monoterpenes are present in free and glycosidcally bound forms. Free monoterpenes directly contribute their flavor scents to grape and wine, whereas bound monoterpenes can be converted into monoterpene aglycones through acid and/or enzymatic hydrolysis during wine-making and aging process. These released monoterpene aglycones can further improve the overall aroma of grape wine with their flavor features (Dimitriadis and Williams, 1984; Gunata et al., 1985; Mandaokar et al., 2006). Compared with free monoterpenes, monoterpenyl glycosides exist in grape berry with a much higher level, indicating that the composition of monoterpenyl glycosides in grape exerts a much more important role in determining the potential aroma of grape wine. The recent studies on the production of free and bound terpenes in grape berries during development stages have reported a dramatic accumulation of terpenesyl glycosides with grape berry maturation (Fenoll et al., 2009; Vilanova et al., 2012; Bönisch et al., 2014a,b). Additionally, our previous study reported a significant difference on the level of glycosidically bound monoterpenes in one mature “Muscat blanc à Petit grain” grape variety cultivated in two wine-making regions with different climates. More importantly, the evolution pattern of these bound monoterpenes was highly accompanied with the expression of a monoterpene glycosyltransferase gene (*VvGT14*), indicating that this enzyme might primarily regulate the production of monoterpenyl glycosides in this variety (Wen et al., 2015).

Three monoterpenol glycosyltransferase genes have been identified to take responsibility of coding *VvGT7* (Bönisch et al., 2014a), *VvGT14* and *VvGT15* (Bönisch et al., 2014b) in *Vitis vinifera* grape varieties. Meanwhile, the concentration ratio of free to glycosidically bound monoterpenes exhibited a significant difference in these different varieties at 15- to 17-week after flowering phase with geraniol and geranyl β-D-glucoside as the dominant terpene metabolites except for *Vitis vinifera* White Riesling (Bönisch et al., 2014a). These indicated that different monoterpenol glycosyltransferase activity and differential glycosyltransferase substrate preferences existed in different grape varieties. A further investigation on the allelic gene and substrate preferences of these glycosyltransferases confirmed the key amino acids of *VvGT7* (Bönisch et al., 2014b). However, some questions remained to be uncovered: (1) How many monoterpenyl glycosyltransferase allelic genes were present in each *Vitis vinifera* variety; (2) which nucleotides of these allelic genes in these varieties exhibited the differences; and (3) If the diversity of glycosyltransferases resulted in the difference on the production of monoterpensyl glycosides.

To better understand the mechanism of monoterpenyl glycoside accumulation in grapes, we selected two *Vitis vinifera* varieties, including “Muscat blanc à Petit grain” and “Gewürztraminer.” “Muscat blanc à Petit grain” variety is considered “Muscat-type” due to high level of monoterpenes, whereas “Aromatic-type” “Gewürztraminer” variety contains moderate level of monoterpenes (Strauss et al., 1986). These two cultivation-limited *Vitis vinifera* varieties were mainly distributed in the northwestern China, and their wines display pleasant floral aroma. It has been reported that “Muscat-type” grapes had linalool, geraniol, citronellol, and nerol as the dominant monoterpene, and the increase on the composition of free volatiles resulted in a concentration accumulation on glycosidically bound volatiles during grape maturation period (Ong and Acree, 1999; Palomo et al., 2006; Vilanova et al., 2012; D'Onofrio et al., 2017). In the present study, we investigated the level of free and bound terpenes in both varieties to establish the profile and potential flavor feature of free and glycosidically bound monoterpenes. More importantly, the abundance of *VvGT7* and *VvGT14* transcripts in both grape varieties were compared with an aim of elucidating the difference on single nucleotide between the *VvGT7* and *VvGT14* alleles, which could further uncover the correlation between the production of monoterpene glycosides and the related gene alleles.

## Materials and methods

### Plant material

*Vitis vinifera* “Muscat blanc à Petit grain” and “Gewürztraminer” grape berries were harvested in a commercial vineyard at GaoTai county, Gansu Province, China (39.14°N, 99.84°E) under 2010 and 2011 vintages. Grapevines for both varieties were arranged in north-south oriented rows with a 2.0-m row space on a sloping trunk with horizontal cordon. The distance between two plants was approximately 1.0 m in each row, and the own-rooted vines were planted in 2001. During the experiment period, pest and disease management and fertilization applied to the studied vineyards were complied with the local wine grape cultivation practices. A single variety vineyard was artificially divided into three plots and each plot was considered a biological replicate. In each plot, 100–150 grape berries were randomly sampled from at least 50 vines at each maturation stage for each variety. These berry samples were placed in Ziploc bags covered with foam ice boxes, and shortly transported to the experimental station close to the vineyard. Afterwards, 50 berries were used for the determination of total soluble solid content and pH value, and the remainder was immediately frozen under liquid nitrogen stored at −80°C for further use.

### Chemicals

Tartaric acid, glucose, methanol, methylene chloride, NaCl, and NaOH were of analytical grade and purchased from Beijing Chemical Works (Beijing, China). D-Gluconic acid lactone, polyvinylpolypyrrolidone (PVPP), and all monoterpene standards were purchased from Sigma-Aldrich (St. Louis, MO, USA). Glycosidase AR2000 was received from DSM Food Specialties Beverage Ingredients (Heerlen, Netherlands). Cleanert PEP-SPE (50 mg/6 mL) and Cleanert ODS-C18 (150 mg/6 mL) were purchased from Bonna-Agela Technologies (Tianjin, China).

### Physicochemical indexes

Grape berries (approximately 50 berries) after removing seeds were squeezed to obtain grape juice. The grape juice was used for the analyses of total soluble solids and pH value. The total soluble solids of the juice were measured using an automatic temperature-compensated digital Refractometer (Pocket Refractometer Pal-1, Atago, Japan), and the results were expressed as °Brix. The pH value was determined using a potentiometric titrator PB-10 (Sartorius, Germany). Each measurement was performed in triplicate.

### Extraction of free and glycosidically bound volatile compounds

Extraction of free and glycosidically bound volatiles from the grape berry samples followed the published methods (Wen et al., 2015; Lan et al., 2016). For each replicate, about 200 grape berries were de-seeded and ground under liquid nitrogen, and then the flesh was mashed and blended. After cold maceration at 4°C for 120 min, the flesh was immediately centrifuged at 6,000 × g at 4°C for 10 min to get clear grape juice. In brief, 5 mL of the grape juice was mixed with 10 μL of 4-methyl-2-pentanol (internal standard) and 1 g NaCl in a 20-mL PTFE-silicon septum capped vial containing a magnetic stirrer. The vial was equilibrated at 40°C for 30 min under a 500-rpm agitation. A 2-cm DVB/CAR/PDMS 50/30 μm solid-phase micro-extraction (SPME) fiber (Supelco, Bellefonte, PA, USA) was activated at 250°C before sample extraction. The activated SPME fiber was inserted into the headspace of vial to adsorb volatiles at 40°C for 30 min under the same agitation. Finally, the SPME fiber was inserted into the GC injector port for 8 min to desorb volatiles.

For extraction of glycosidically bound volatiles, Cleanert PEP-SPE cartridges (Bonna-agela Technologies, China, 200 mg/6 mL) were pre-conditioned using 10 mL of methanol and then 10 ml of Mili-Q water. The grape juice (5 mL) was loaded onto the pre-conditioned Cleanert PEP-SPE cartridges. Water-soluble compounds were eluted with 5 mL of Mili-Q water, whereas 10 mL of dichloromethane was washed through the cartridge to remove the free volatiles. Bound volatile precursors were finally eluted from the cartridges using 20 mL of methanol with a flow rate at 2 mL/min. The resultant methanol eluate was evaporated to dryness under a rotary evaporator under and then re-dissolved in 10 mL of 2 mol/L citrate-phosphate buffer solution (pH 5.0). Subsequently, 100 μL of the AR2000 solution (100 mg/mL in 2 mol/L citrate-phosphate buffer, pH 5.0) was added to the extract. The resultant mixture was sealed and vortexed, and then placed in an incubator at 40°C for 16 h to release bound volatile aglycones. The released aglycones were extracted using the same SPME method mentioned above.

### GC-MS

An Agilent 7890N gas chromatography with an autosampler system, coupled with an Agilent 5975C mass spectrometer (Agilent Technologies, Santa Clara, CA, USA), was used to analyze volatile compounds in these grape varieties. The volatiles were separated on an HP-INNOWAX capillary column (60 m × 0.25 mm id, 0.25 μm film thickness) (J&W Scientific, Folsom, CA, USA) under a carrier gas (helium) flow rate of 1 mL/min. The temperature gradient on the oven was programmed as follows: 50°C (1 min hold) to 220°C at 3°C/min and held at 220°C for 5 min. The ion source on mass spectrometer was set at 230°C with the interface at 280°C. A full scan mode from *m/z* 30 to 350 was used. A C6-C24 n-alkane series (Supelco, Bellefonte, PA, USA) under the same chromatographic condition was used to calculate retention indices. Volatiles with the available standard were identified by matching their mass spectrum with the Standard NIST05 library and retention indices of the reference standard. For volatiles without the available standard, they were tentatively identified by comparing their mass spectrum with the Standard NIST05 library and the retention indices reported in the literature. For quantification, the volatiles with the reference standard were quantified using peak area ratio of standard to internal standard vs. reference standard concentration, whereas the quantitation of the volatiles without the available standard was carried out using the standard with the similar carbon atoms or structure. The detailed information was listed in Table S1.

### RNA extraction and quantitative real-time PCR analysis

For each biological replicate, 10 berries without seeds were ground into powder and 1 g powder was used for RNA extraction. Total RNA was extracted using a Plant RNA Isolation Kit (Sigma RT-250, St. Louis, MO, USA), whereas RNA integrity was verified using agarose gel electrophoresis. The RNA quantity and quality were evaluated using a Qubit 2.0 Fluorometer RNA Assay Kit (Invitrogen Inc., Massachusetts, USA) and an Agilent 2100 Bioanalyzer RNA 6000 Nano Kit (Agilent Technologies, Santa Clara, CA, USA), respectively. Additionally, 5 mg total RNA was used to synthesize first strand complementary DNA (cDNA) using SuperScript First-Strand Synthesis System (Promaga, Madison, WI, USA). Gene specific oligonucleotide primer for *VvGT7, VvGT14*, and *VvGT15* were designed by the Primer-Blast from NCBI (https://www.ncbi.nlm.nih.gov/tools/primer-blast/) and their sequences are listed in Table S2. Three grapevine reference genes were used for coding GAPHDH (CB975242), actin (EC969944), and ubiquitin (EC929411). Additionally, 10 μL of SYBR®Premix Ex TaqTM, 0.5 μL of ROX Reference Dye (50×) (Takara, Dalian, China), 1 μL of 10 mM primer mixture (forward primer and reverse primer), 4 μL of diluted template cDNA, and 4.5 μL of ddH_2_O were mixed to generate PCR solution. During PCR cycling program, an initial denaturation was carried out at 95°C for 30 s, followed by 40 cycles of amplification at 94°C for 10 s, and then at 60°C for 31 s. Melt curve analysis from 65 to 95°C was used to detect possible primer dimers or nonspecific amplification in the cDNA samples. The specificity of primers was confirmed using agarose gel electrophoresis. The expression level of target gene was calculated using the equation below,

The expression level = 2^−ΔCT^

$$\Delta CT=CT^{target}-C^{Tref}$$

Where *C*^*Tref*^ represents geometric mean of three reference gene threshold cycles (CTs). The mean and standard deviation were estimated after 2^−ΔCT^ calculations. The q-PCR reaction for each biological replicate was carried out in triplicate.

### Comparative sequencing

Total RNA extracted from the mature grape berries was used as template due to the high expression of *VvGT7* and *VvGT14*. Afterwards, AMV reverse transcriptase (Promega, Madison, WI, USA) and oligo d(T)18 (Takara, Dalian, Liaoning Province, China) were used to synthesize cDNA regarding the instructions of the manufacturer. The full-length nucleotide sequence cloning on the gene coding region, using Master Mix (ThermoFisher Scientific, MA, USA), was carried out in high-fidelity buffer under a PCR condition as follows: 94°C for 30 s, followed by 30 cycles consisting of 94°C for 30 s, 62°C for 60 s, and 72°C for 90 s, and then a final elongation step of 72°C for 5 min. Subsequently, the PCR products were purified using Wizard SV Gel under PCR Clean-Up System (Takara, Dalian, Liaoning Province, China). The purified PCR products were further A-tailed using Taq DNA Polymerase (ThermoFisher Scientific, MA, USA). Afterwards, the A-tailed PCR products were ligated into pMD-19 vector (Promega, Madison, WI, USA), and the sequencing was performed by Sangon (Shanghai, China).

### Statistical analysis

Data were expressed as the mean ± standard deviation. One-way analysis of variance was carried out to determine the significance of the mean value under Duncan's multiple range tests at a significant level of 0.05 (Vilanova et al., 2009). Hierarchical cluster analysis was performed using MetaboAnalyst 2.0 (http://www.metaboanalyst.ca/) through “Time Series Analysis” interfaces. Nucleotide sequences were assembled from DNASTAR (http://www.dnastar.com/).

## Results and discussion

### Evolution of total soluble solids and pH value in grape berries during development stages

A significant difference on the total soluble solids was observed in these two grape varieties (Table 1). More importantly, each variety under these two vintages also exhibited the significant differences on their total soluble solids. It should be noted that the greatest accumulation of the total soluble solids occurred at the beginning of the maturation stage, which was consistent with the previous reports (Vilanova et al., 2009; Gomez et al., 2010). Similar as the total soluble solids, these two grape varieties also exhibited an increase on their pH value with the berry development stages.

**Table 1:** Physicochemical indexes of two *Vitis vinifera* varieties at harvest.

**Days after flowering**	**“Muscat blanc à Petit grain”**				**“Gewürztraminer”**			
	**Brix**		**pH value**		**Brix**		**pH value**	
	**2010**	**2011**	**2010**	**2011**	**2010**	**2011**	**2010**	**2011**
30	3.70 ± 0.04^a^	4.75 ± 0.00^b^	2.66 ± 0.00_a_	2.53 ± 0.10_a_	3.75 ± 0.04^a^	4.50 ± 0.00^b^	2.56 ± 0.09_a_	2.57 ± 0.02_a_
42	5.25 ± 0.00^a^	7.08 ± 0.90^b^	2.62 ± 0.10_a_	2.56 ± 0.00_a_	4.80 ± 0.07^a^	6.85 ± 0.07^b^	2.65 ± 0.05_a_	2.61 ± 0.00_a_
57	10.45 ± 0.30^b^	12.03 ± 0.20^b^	2.69 ± 0.00_a_	2.68 ± 0.00_a_	8.15 ± 0.11^a^	13.95 ± 0.08^d^	2.91 ± 0.15_a_	2.91 ± 0.00_a_
72	15.10 ± 0.20^a^	14.80 ± 1.70^a^	3.01 ± 0.10_a_	3.12 ± 0.70_a_	15.85 ± 0.18^a^	17.50 ± 0.00^b^	3.01 ± 0.07_a_	3.16 ± 0.00_a_
86	17.90 ± 0.50^a^	20.65 ± 0.00^b^	3.10 ± 0.00_a_	3.32 ± 0.20_b_	19.75 ± 0.18^b^	21.25 ± 0.05^d^	3.01 ± 0.01_a_	3.15 ± 0.00_ab_
100	16.45 ± 0.70^a^	18.50 ± 0.20^b^	3.15 ± 0.50_a_	3.33 ± 0.00_b_	22.50 ± 0.35^b^	18.35 ± 0.07^b^	3.15 ± 0.01_a_	3.11 ± 0.02_a_
106	18.40 ± 0.60^a^	25.95 ± 0.00^b^	3.36 ± 0.70_a_	3.40 ± 0.30_a_	27.55 ± 0.39^b^	25.75 ± 0.21^b^	3.34 ± 0.03_a_	3.46 ± 0.05_a_

*Data are mean ± standard deviation of triplicate tests. Different upper letters in each category represent significant differences of Brix between different varieties under each berry development stages at p ≤ 0.05. Different lower letters represent significant differences of pH value at each development stage for each variety at p ≤ 0.05*.

### Free and glycosidically bound monoterpene profile

**Figure 1:**
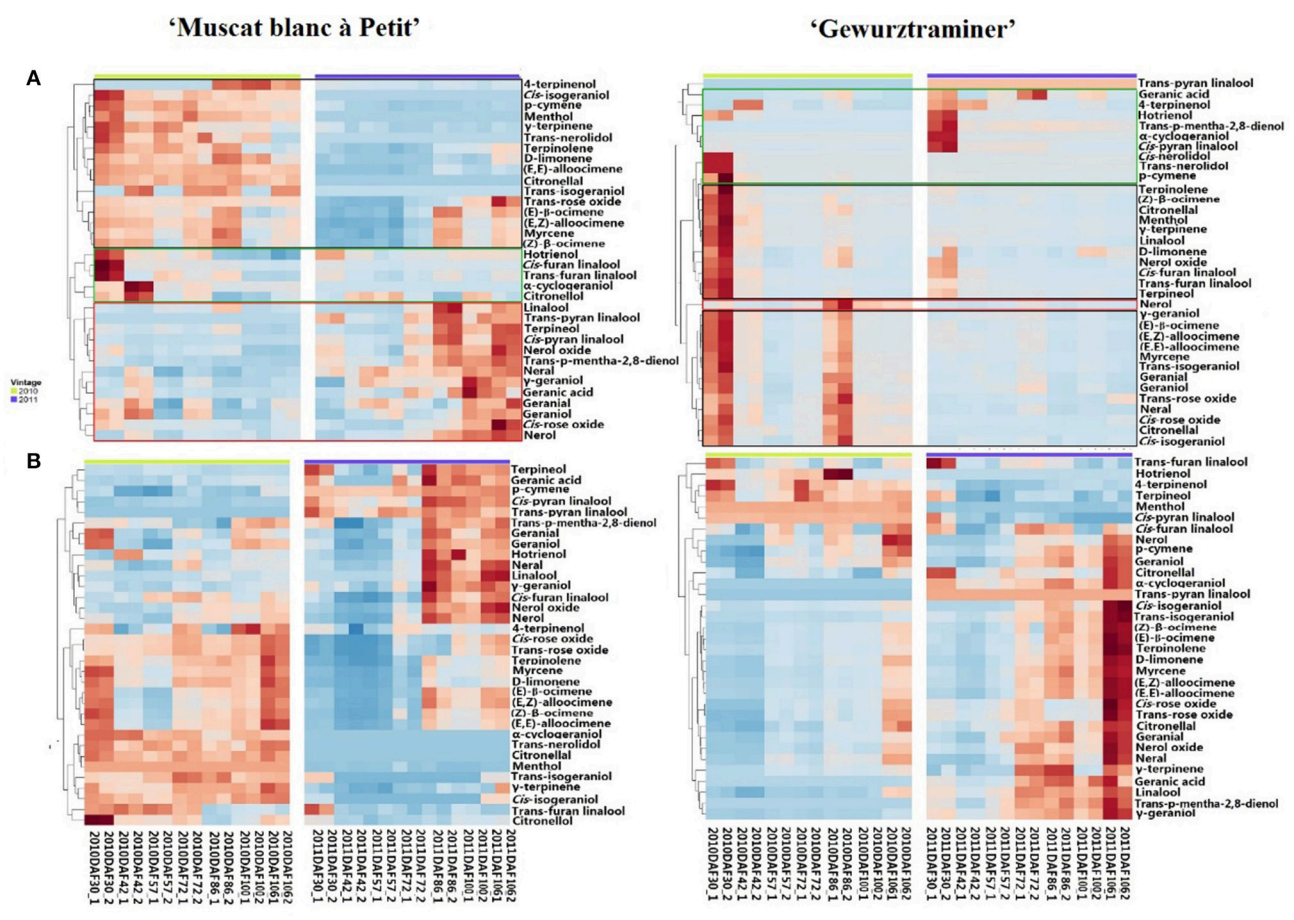
Hierarchical cluster analysis of monoterpenes in developing grapes in 2010 and 2011. Green block represents Cluster I, Black one represents Cluster II and red one represents Cluster III. **(A)** Free-form monoterpenes in the berries of “Muscat Blanc a Petits Grains” and “Gewürztraminer.” **(B)** Monoterpenyl glycosides in the berries of “Muscat Blanc a Petits Grains” and “Gewürztraminer.”

A total 35 free monterpenes were identified in this study, and the evolution of these free monterpenes in both varieties during the berry development stage was visualized using hierarchical cluster analysis (Figure 1A). For each grape variety, these free monoterpenes exhibited a similar evolution trend in the 2010 and 2011 vintages although the variation was observed on their concentration. For instance, the concentration of most the free monoterpenes in the grape variety “Gewürztraminer” was higher in the 2010 vintage rather than the 2011 vintage. Additionally, these free monoterpenes displayed three evolution patterns during the grape development phases. The free monoterpenes in the first evolution pattern decreased on their concentration in the berries with the berry maturation (Black Block in Figure 1A). In the second evolution patter, the free monoterpenes reached their concentration peak at the early development stage and veraison, followed by a concentration decrease in the berries at the late stage of the berry development (Green Block in Figure 1A). A concentration increase on the free monoterpenes during the berry maturation period was observed as the third evolution pattern (Red Block in Figure 1A). It was observed that some free monoterpenes displayed the different evolution patterns in these two grape varieties during their development stages. For example, *cis*-furan linalool and *trans*-furan linalool exhibited the first evolution pattern in the variety “Muscat blanc à Petit grain.” However, these two volatiles followed the second evolution pattern in the variety “Gewürztraminer.” In addition, the free *cis*-pyran linalool exhibited the third and the first evolution pattern in the variety “Muscat blanc à Petit grain” and “Gewürztraminer,” respectively. It was also observed that the free hotrienol in both varieties followed the first evolution pattern with the berry development stages, whereas the third evolution pattern took place for nerol in both varieties. Besides, the free monoterpenes that had the similar evolution patter as nerol in the variety “Muscat blanc à Petit grain” included *cis*-rose oxide, geraniol, neral, nerol oxide, terpinerol, linalool, *trans*-p-mentha-2,8-dienol, and *trans*-pyran linalool. We speculated that the difference on these free monoterpenes with the third evolution pattern resulted in the differentiation on the overall aroma features of these two grape varieties since these volatiles in grape exhibited a concentration elevation with the berry maturation (Ong and Acree, 1999; Palomo et al., 2006).

Different as the free-form monoterpenens, almost all the glycosidically bound monoterpenes showed an increase on their concentration in both varieties during the development period, and their concentration reached to the highest at the harvest stage (Figure 1B). Furthermore, the accumulation of these glycosidically bound monoterpenes mainly happened at the beginning of the maturation stage in the variety “Muscat blanc à Petit grain.” whereas the significant concentration elevation of these bound volatiles in the variety “Gewürztraminer” was found to be the late stage of the maturation. It should be noted that citronellol glucoside reached its highest concentration at the early maturation stage in the variety “Muscat blanc à Petit grain” and in the late development stage of the variety “Gewürztraminer,” respectively. Besides, *trans*-furan linalool glucoside exhibited the highest level in these varieties at the early stage of the development, followed by a concentration reduction with the berry maturation.

### Key free and glycosidically bound monoterpenes

Free volatiles are considered the direct sensory contributor to the overall aroma of grape and wine, and its sensory contribution can be estimated using odor activity value (OAV) (Schieberle, 1991). The odor activity value of a free volatile is calculated by its concentration in fruits over its sensory threshold in water. It has been accepted that a volatile with its OAV above 1 can serve as a key odorant in bringing its featured flavor to the overall aroma of fruits (Guth, 1997). In the present study, the free monoterpenes that had its concentration above their sensory threshold included *cis*-rose oxide, geraniol, *D*-limonene, lianalool, myrcene, and nerol (Table 2). These six monoterpenes have been reported to exhibit the floral note (Bayrak and Akgül, 1994; Luan et al., 2005). Linalool appeared to be the critical terpene that significantly determines the floral attribute of the variety “Muscat blanc à Petit grain” due to its extremely high OAV. Additionally, geraniol also played an important role in the aroma of the variety “Muscat blanc à Petit grain.” In the variety “Gewürztraminer,” these six free monoterpenes showed the similar OAV value, indicating that the floral aroma of this variety resulted from the combined contribution of these free monoterpenes. Our results were similar as the previous studies where linalool, *cis*-rose, geraniol and other terpenes together established the canned lychee flavor feature for the variety “Gewürztraminer,” whereas linalool and geraniol were the key terpenes for the floral and citric odors of the variety “Muscat blanc à Petit grain” (Ong and Acree, 1999; Palomo et al., 2006).

**Table 2:** Odor thresholds and odor activity values (OAVs) of free monoterpenes in two *Vitis vinifera* varieties at harvest.

**Free monoterpenes**	**Odor thresholds (μg/L)**	**Discription**	**OAV (“Muscat blanc à Petit grain”)**		**OAV (“Gewürztraminer”)**	
			**2010**	**2011**	**2010**	**2011**
**myrcene**	**15^a^**	**Floral**	**1.42**	**1.62**	**2.96**	**0.59**
**limonene**	**10^b^**	**Floral**	**1.76**	**1.98**	**1.33**	**3.17**
**terpinolene**	200	–	0.036	0.059	0.0036	0.0089
***cis*****-rose oxide**	**0.5^b^**	**Rose**	**1.17**	**4.25**	**3.59**	**3.23**
***trans-*****rose oxide**	**0.5^b^**	**Rose**	**0.74**	**1.27**	**1.44**	**2.27**
nerol oxide	100	–	0.011	0.073	0.016	0.024
**linalool**	**6^a^**	**Floral, Lavender**	**11.03**	**51.54**	**6.80**	**3.07**
4-terpinenol	130	Floral	6	0.0038	0.00013	0.00086
neral	30^a^	–	0.012	0.038	0.10	0.021
terpineol	330	–	0.013	0.097	0.0042	0.0063
geranial	32	–	0.087	0.15	0.48	0.087
citronellol	40^b^	Grass, Rose	0.11	0.098	0.34	0.079
γ-geraniol	40	Floral	0.015	0.045	0.099	0.021
nerol	300^b^	Citrus, Floral	0.025	0.089	0.18	0.035
*trans-*isogeraniol	40	–	0.0012	0	0.021	0.0029
**geraniol**	**40^a^**	**Rose, Lemon**	**2.83**	**4.78**	**5.63**	**1.91**

*Odor and Flavour Detection Thresholds in Water (In Parts per Billion, lg/L) (http://www.leffingwell.com/odorback.htm)*.

a Pino and Mesa, 2006;

b *Fenoll et al., 2009*.

*Bold font indicates that the compound is odoriferous and its concentration reaches or exceeds its perception threshold*.

Monoterpenyl glycosides are the potential flavor contributor in grapes since these volatiles can be hydrolyzed into monoterpene aglycones and sugar moiety under acid and/or enzymatic hydrolysis during wine fermentation and aging process (Gunata et al., 1985). The released monoterpene aglycones can further incorporate their sensory features to the aroma of wine. Their potential aromatic contribution can be estimated using their odor activity value, and monoterpenyl glycosides with their OAV above 1 could be considered a key potential volatile for the wine sensory attribute (Guth, 1997). In the present study, both varieties showed 10 glycosidically bound monoterpenes with their OAV above 1 (Table 3). Among them, linalool glucoside, geraniol glucoside, and *cis*-rose oxide glucoside played the essential role in enhancing the floral odor of the wine made of the variety “Muscat blanc à Petit grain.” The potential contribution to the floral aroma of the “Gewürztraminer” wine was mainly derived from the hydrolysis of the bound geraniol, *cis*-rose oxide, myrcene, and limonene.

**Table 3:** Odor thresholds and potential odor activity values (OAVs) of monoterpenyl glucosides in *Vitis vinifera* varieties at harvest.

**Bound monoterpenes**	**Odor thresholds (μg/L)**	**Discription**	**OAV (“Muscat blanc à Petit grain”)**		**OAV (“Gewürztraminer”)**	
			**2010**	**2011**	**2010**	**2011**
**myrcene**	**15^a^**	**Floral**	**3.58**	**2.74**	**11.67**	**28.01**
**limonene**	**10^b^**	**Floral**	**4.22**	**2.88**	**11.59**	**22.13**
terpinolene	200	–	0.052	0.042	0.054	0.19
***cis*****-rose oxide**	**0.5^b^**	**Rose**	**9.46**	**9.39**	**12.26**	**22.10**
***trans-*****rose oxide**	**0.5^b^**	**Rose**	**4.46**	**4.16**	**5.52**	**9.17**
nerol oxide	100	–	0.059	0.12	0.089	0.19
**linalool**	**6^a^**	**Floral, Lavender**	**7.66**	**94.11**	**3.35**	**5.67**
4-terpinenol	130	Floral	0.0051	0.0037	0.00084	0.0014
neral	30^a^	–	0.29	0.63	1.19	1.92
Terpineol	330	–	0.012	0.022	0.021	0.013
geranial	32	–	0.55	0.78	2.35	5.83
Citronellol	40^b^	Grass, Rose	0.36	0.32	0.87	1.46
γ-geraniol	40	Floral	0.099	0.19	0.33	1.95
**nerol**	**300^b^**	**Citrus, Floral**	**1.70**	**3.32**	**3.11**	**2.37**
*trans-*isogeraniol	40	–	0.0086	0.18	0.079	0.59
**geraniol**	**40^a^**	**Rose, Lemon**	**9.58**	**10.17**	**22.13**	**30.17**

*Odor and Flavour Detection Thresholds in Water (In Parts per Billion, lg/L) (http://www.leffingwell.com/odorback.htm)*.

a Pino and Mesa, 2006;

b *Fenoll et al., 2009. Bold font indicates that the compound is odoriferous and its concentration reaches or exceeds its perception threshold*.

**Figure 2:**
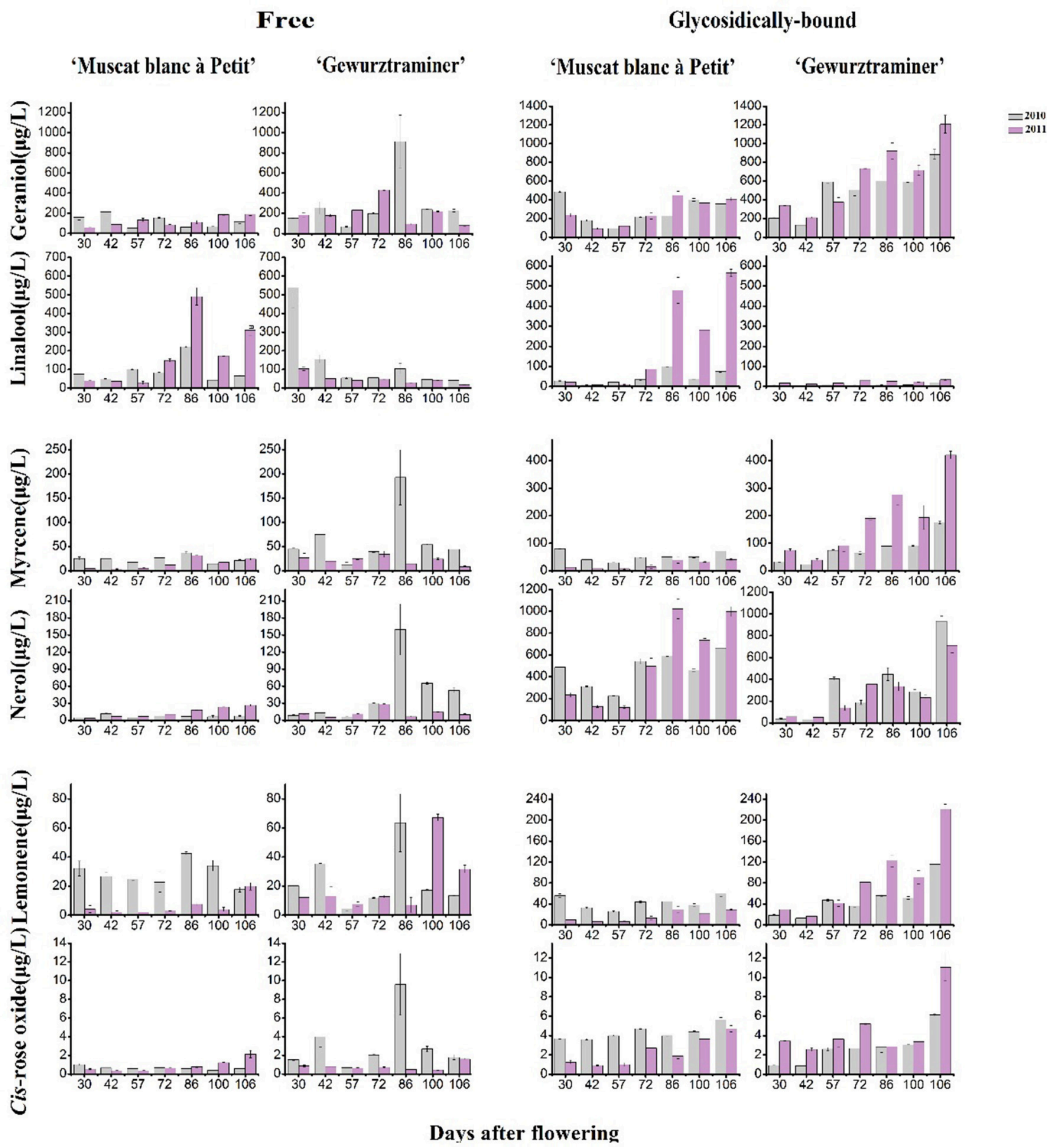
Evolution of the concentrations of six main monoterpenes during berry development.

Figure 2 shows the evolution pattern of six monoterpenes in their free and glycosidically bound forms in both varieties during the grape development period. These monoterpenes represented 67–85% of the total terpene concentration in these varieties and they exhibited the high odor activity value. It was observed that the evolution patterns of the free monoterpenes varied with both the varieties and vintages. They appeared to be interfered with the vintage more significantly. These six free monoterpenes all had a sudden concentration increase on the 86th day after flowering in the variety “Gewürztraminer” under the 2010 vintage, which was considered an abnormal phenomenon, probably because the samples lack of representative were used for this detection. With regard to the bound monoterpenes, they all showed an overall increasing trend on their concentration in both varieties during the beginning of veraison to harvest (Figure 2). Moreover, this evolution pattern was not altered with the vintage in this study, but the concentration of their respective compounds obviously differed between both years. Compared with the vintage 2010, most bound monterpenes showed higher concentration in the vintage 2011 in the variety “Gewürztraminer.” The previous studies reported that most of bound monoterpenes were accumulated to the highest concentration in grapes at the ripening stage (Dimitriadis and Williams, 1984; Gunata et al., 1985; Wilson, 1986). It has been accepted that free monoterpenes could be easily affected by the alteration of climates during berry development stages due to their volatile property. Bound monoterpene composition, however, could be more suitable to represent the variety feature since the sugar moiety decreases their volatility (Wirth et al., 2001). In the variety “Gewürztraminer,” geraniol glucoside appeared to be the highest monoterpenly glycoside at harvest, followed by the nerol and myrcene glycoside. But in the ripening “Muscat blanc à Petit grain” grapes, the bound linalool was the primarily monoterpenyl glycoside. The glycosidic geraniol and nerol also showed the high level in this variety at harvest.

### Comparison on free to glycosidically bound monoterpene and *VvGTs* expression

**Figure 3:**
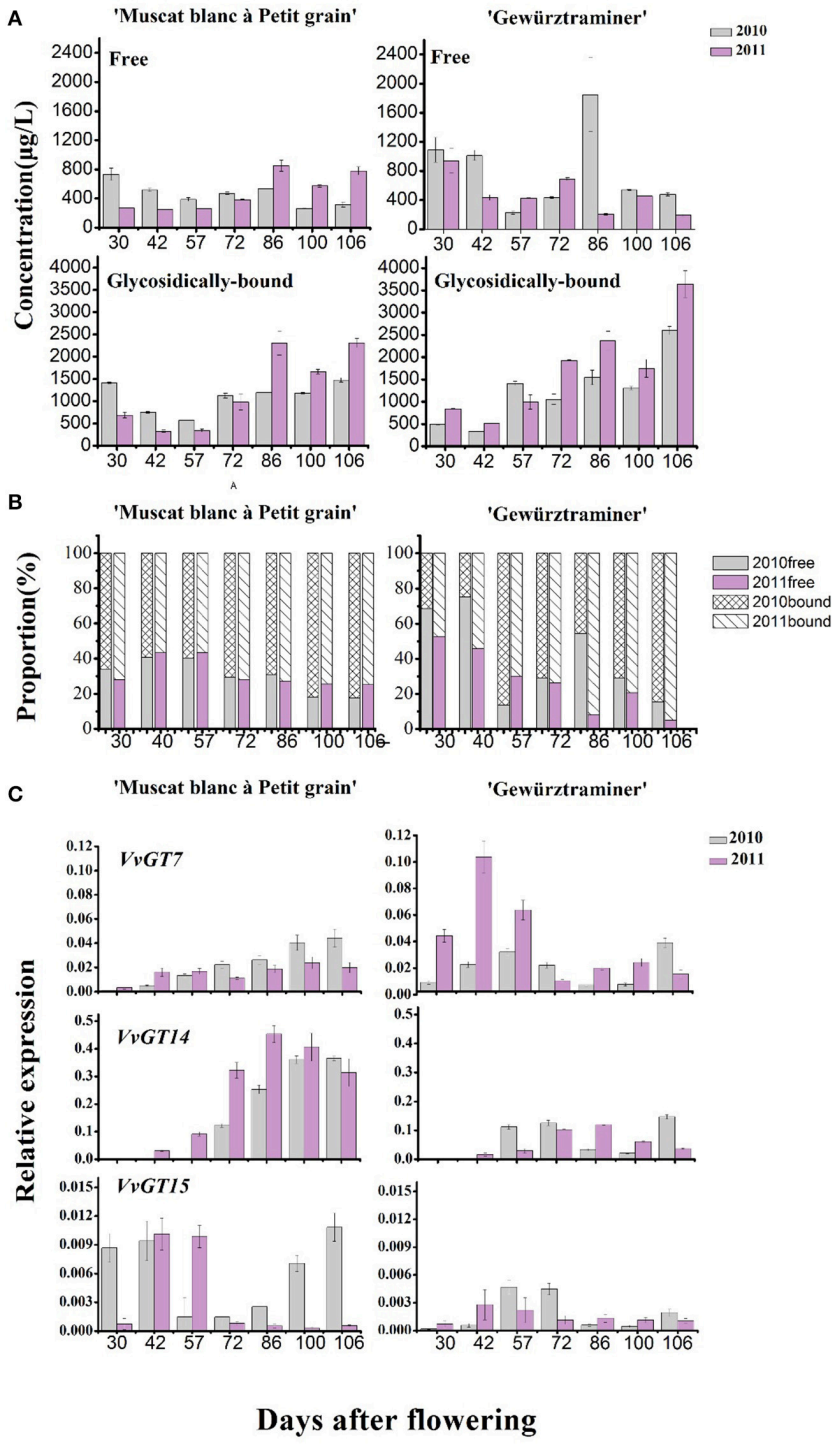
**(A)** Changes of the total concentrations of free-form (first line) and glycosidically bound (second line) monoterpenes along with grape berry maturation. **(B)** The percentage of free and glycosidically-bound concentrations, respectively, to the total. **(C)** Relative expression amount of three genes coding for monoterpenyl glycosyltransferases in developing “Muscat blanc à Petit” and “Gewurztraminer” berries in 2010 and 2011.

The concentration of the total free monoterpenes was higher in the early development stage (“green fruit” stage) in both varieties, which mainly was attributed to the presence of the grape flower monoterpenes (Matarese et al., 2014). Afterwards, the fruit expansion stage resulted in the concentration decrease of the total free monoterpenes in these varieties. An increase on the concentration of the total free monoterpenes was observed at the starting of the veraison stage, followed by a concentration decrease at the end of the veraison (Figure 3A). It should be noted that the total free monoterpene concentration only increased during 7–10 days before harvest in the variety “Muscat blanc à Petit grain.” In terms of the bound monoterpenes, their total concentration showed an overall continuous increase since the véraison stage in both varieties, and the concentration was much higher in the 2011 vintage. It has been reported that the accumulation of glycosidically bound terpenes was positively related to the level of total soluble solids in grapes (Battilana et al., 2011). This explained the present observations that the bound monoterpenes level elevated along with grape berry ripening and both varieties possessed higher level of the glycosidic monoterpenes under the 2011 vintage, which corresponded to the increasing soluble solids. But from the view of the vintage, it was not always that the higher total soluble solids the higher bound monoterpenes (Table 1, Figure 3A), suggesting that in addition to sugar level, many other factors, such as the vintage climate, also markedly interfere with the production of the bound monoterpenes.

To understand the effect of vintage climate on the monoterpene synthesis, we summed up the data of rainfall, sunshine hours, daily average temperature and diurnal temperature varaition at the experimental site in the 2 years, according to the sampling time (Table 4). Compared to the year 2011, more rainfall and less sunshine hours happened in the grape growing season of 2010, particularly in 1 month before the harvest (72nd–106th day after flowering), which could result in a lower accumulation of glycosidically bound monterpenes in vintage 2010 in the two varieties (Figure 3). Slight difference in the daily average temperature appeared throughout berry development in the 2 years. Although no reports have been documented about the effect of rainfall amount on the accumulation of terpenes in grapes during development window, it was accepted that the difference on the rainfall period was accompanied by an alteration in both sunshine intensity and air temperature. Such an alteration could indirectly affect the biosynthesis of volatiles in grapes (Bureau, 2000; Zhang et al., 2014). For example, reducing the light intensity during grape development stage has been reported to cause a content reduction on terpenes in grapes at harvest (Belancic et al., 1997). In addition, free linalool has been reported to be reduced in its concentration in grapes under a shading treatment since this terpene was sensitive to light (Zhang et al., 2014).

**Table 4:** The meteorological index in the experimental site in 2010 and 2011.

**Days after flowering**	**Rainfall(mm)**		**Dialy average temperature(°C)**		**Temperature difference between day and night(°C)**		**Sunshine duration (hour)**	
	**2010**	**2011**	**2010**	**2011**	**2010**	**2011**	**2010**	**2011**
30–42	10	51	26.68	26.57	16.13	16.28	159.2	156.2
42–57	0	9	25.39	24.17	12.62	16.27	126.3	143.3
57–72	0	488	22.17	20.51	16.79	11.77	159.2	102.9
72–86	171	30	18.59	20.80	13.79	13.56	128.7	125.00
86–100	585	34	16.60	14.75	11.55	14.08	82.1	128.3
100–106	0	0	13.86	14.98	10.93	16.13	39.0	56.4

*“30–42” indicates the 30th to 42nd day after flowering, so are the rest*.

Figure 3B illustrated that the glycosidically bound monoterpene concentration percentage gradually increased in both varieties after the veraison stage (57 days after flowering), whereas a decrease was found in the free monoterpene concentration percentage. This indicated that monoterpenyl glycosides were accumulated in grapes more rapidly than their free forms in berry ripening period. Higher percentage on monoterpenyl glycoside was found in the ripen “Gewürztraminer” variety under both vintages. It has been confirmed that free monoterpenes can be converted into glycosidically bound form under the activity of monoterpenyl glycosyltransferases in grapes (Lücker et al., 2004; Martin et al., 2012). So far, three monoterpenyl glycosyltransferases in *Vitis vinifera* grapes have been identified and characterized, including *VvGT7, VvGT14*, and *VvGT15* (Bönisch et al., 2014a,b). Besides, geraniol, nerol, and citronellol have been reported to be the common substrates for these three enzymes, whereas *VvGT14* can also convert free linalool into linalool glycoside (Bönisch et al., 2014b). In the present study, the transcript abundance of *VvGT15* was very low in both varieties in the whole development period (Figure 3C), which is some different with the formers report (Castellarin et al., 2007). It should be worth noting that *VvGT14* exhibited a high transcript amount particularly in the variety “Muscat blanc à Petit grain” during the stage of veraison to harvest. The expression of *VvGT14* remained in a high level since the veraison in this variety under both vintages. This might result in the concentration increase on the total monoterpenyl glycosides and individual glycosidically bound monoterpenes (such as the bound linalool, geraniol, and nerol) in this period (Figure 3C). Similarly, the relation between the expression of *VvGT14* and the accumulation of the bound monoterpenes was also found in the variety “Gewürztraminer” during the development. For example, the dramatic accumulation on the bound geraniol and nerol in the variety “Gewürztraminer” at the period of 42- to 57-day and 100- to 106-day of the 2010 vintage, respectively, were accompanied with the increase on the *VvGT14* transcript at the same development period (Figure 2B). In 2011, the significant accumulation of the *VvGT14* transcript and the monoterpenyl glycosides occurred during the same development period (57- to 72-day) in the variety “Gewürztraminer.” Besides, *VvGT7* was highly expressed at the early stage of the variety “Gewürztraminer” development, which might also cause the concentration increase of the bound geraniol and nerol in this stage. Such a relation was not found between the *VvGT7* expression and the production of the bound geraniol and nerol in the variety “Muscat blanc à Petit grain” under the berry maturation period of both vintages (Figure 3).

### Single nucleotide polymorphism of *VvGT14* and *VvGT7*

**Figure 4:**
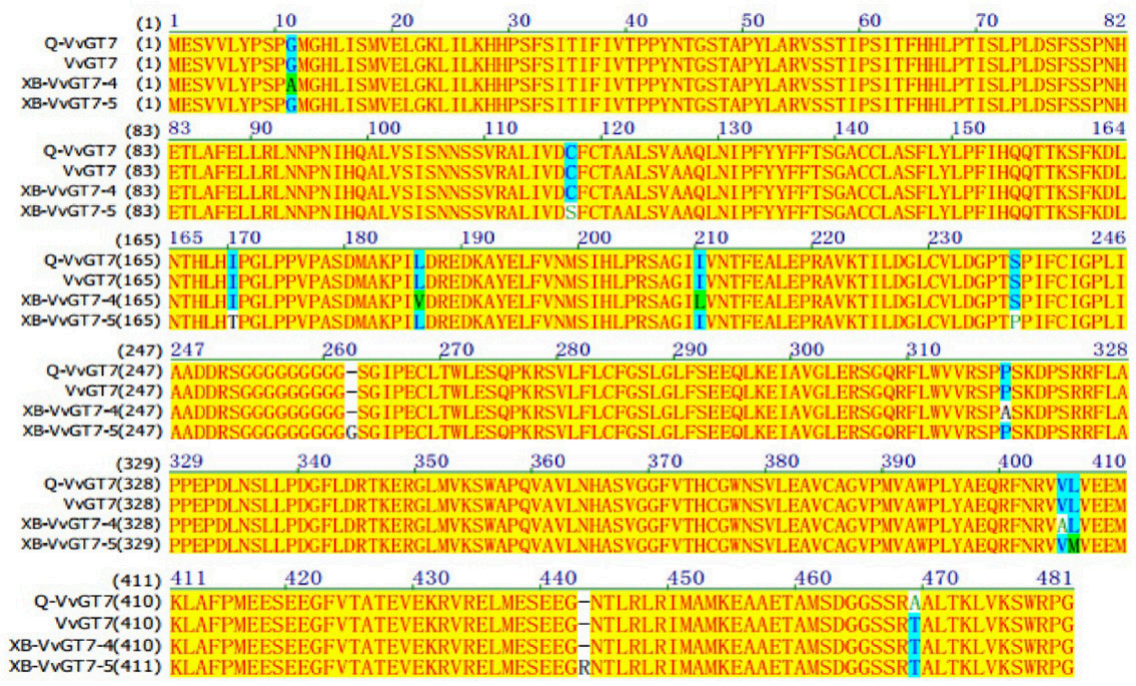
Amino acid sequence alignment of VvGT7 from “Gewürztraminer” and “Muscat blanc à Petit grain” grape berries. Q-VvGT7 represents the VvGT7 sequence of “Gewürztraminer” grapes; XB-VvGT7-4 and XB-VvGT7-5 represent the sequences of two VvGT7 alleles from “Muscat blanc à Petit grain” grapes, VvGT7 represents the sequence from “Pinot noir” grapes published in NCBI.

From the results above, we speculated that it was *VvGT14* and *VvGT7*, instead of *VvGT15*, that played the essential role in producing monoterpenyl glycosides in both varieties. The critical amino acids have been reported in the previous studies in the sequence of *VvGT7* and *VvGT14* (Bönisch et al., 2014a,b). In the present study, the full-length nucleotide sequence of *VvGT14* and *VvGT7* were isolated in both varieties to determine the alleles of these two enzymes. It was observed that the variety “Gewürztraminer” only contained one *VvGT7* allele (*Q-VvGT7*), whereas two *VvGT7* alleles (*XB-VvGT7-4* and *XB-VvGT7-5*) were present in the variety “Muscat blanc à Petit grain” (Figure 4). Additionally, a total of 11 amino acid differences were found in these three allelic proteins with the key position 186, 210, and 318. Regarding the *Q-VvGT7* predicted amino acid sequence, these key residues were 186 leucine (L)- 220 isoleucine (I)- 318 proline (P). It has been confirmed that these three amino acids played the essential role in attaching sugar moiety to the free nerol, citronella, geraniol, and eugenol (Bönisch et al., 2014a). This indicated that the *Q-VvGT7* protein was active for glycosylation of free monoterpenes. A similar allozyme *VvGT7g* was also found in the variety “Gewurztraminer” to glycosylate nerol, citronellol, and geraniol from a previous study (Bönisch et al., 2014a). However, the glycosylation residue in the *XB-VvGT7-4* was valine (V) 186 -leucine (L) 220-alanine (A) 318. This triple mutation (V186L-L210I-A318P) has been reported to decrease the glycosylation conversion ability of nerol, citronellol, and geraniol (Bönisch et al., 2014a). The predicted protein of *XB-VvGT7-5* isolated from the variety “Muscat blanc à Petit grain” had a similar 186 leucine (L)- 220 isoleucine (I)- 318 proline (P) sequence as the variety “Gewürztraminer.” However, the additional arginine was inserted at the position 443 (R) of the *XB-VvGT7-5* sequence. The residue at the position 443 has been reported to be part of the two C-terminals ɑ-helices, and these helices stabilized the spatial structure of the cleft (Wang, 2009). The insertion of arginine at the position 443 might rearrange the catalytic center of the *XB-VvGT7-5* enzyme, influencing its enzyme activity (Bönisch et al., 2014a). Therefore, we speculated that the production of nerol, citronellol, and geraniol glycosides in the variety “Muscat blanc à Petit grain” might not result from *XB-VvGT7-4* or *XB-VvGT7-5*. A previous study reported that the “Muscat a Petits Grains Blancs” FR 90 had two inactive *VvGT7* allozymes due to double mutation of active residues (*VvGT7i* and *VvGT7j*) and one active allozyme (*VvGT7a*) (Bönisch et al., 2014a). Meanwhile, both *VvGT7i* and *VvGT7j* were structurally different due to the insertion of one amino acid (Arg-443). In the present study, the variety “Muscat blanc à Petit grain” was a self-rooted vine, which might have different clones than the “Muscat a Petits Grains Blancs” FR 90.

**Figure 5:**
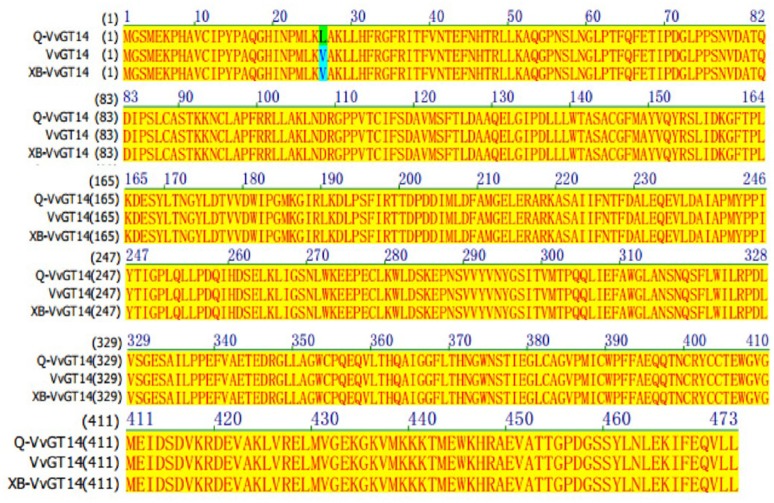
Amino acid sequence alignment of VvGT14 in “Gewürztraminer” and “Muscat blanc à Petit grain” for grape berries. Q-VvGT14 represents the VvGT14 sequence of “Gewürztraminer” grapes; XB-VvGT14 represents the VvGT14 sequence from “Muscat blanc à Petit grain” grapes, VvGT14 represents the sequence from “Pinot noir” grapes published in NCBI.

Only one genotype of *VvGT14* was found in both varieties and these two enzymes (*Q-VvGT14* and *XB-VvGT14*) showed the high similarity on their predicted amino acid sequence (Figure 5). Both sequences contained proline (P) and the existence of 21 amino acid at the position 391 and 165, respectively. This was highly similar as the active *VvGT14a* allele reported in the previous study (Hughes and Hughes, 2009). Proline at the position 391 was part of PSPG motif, and PSPG motif has been reported to be critical for secondary metabolite glycosyltransferase in plant (Hughes and Hughes, 2009). Only one amino acid difference at the position 26 was observed in the amino acid sequence of *XB-VvGT14* and *Q-VvGT14* (valine in *XB-VvGT14* vs. leucine in *Q-VvGT14*). This position was not an active region of enzyme and the different residues in this position did not affect the activity of enzyme (Osmani et al., 2009). Based on the above discussion, we speculated that *XB-VvGT14* and *Q-VvGT14* could provide glycosylation activity to monoterpenes, such as linalool, nerol, and geraniol.

## Conclusion

In conclusion, free monoterpenes in both varieties showed three evolution patterns during berry maturation period under two vintages. An increasing trend on glycosidically bound monoterpenes was observed in both varieties during berry development of two vintages. The accumulation of glycosidically bound monoterpenes in both varieties resulted from the high expression of *VvGT7* and *VvGT14*. Only one *VvGT7* allele (*Q-VvGT7*) was found in the variety “Gewürztraminer” and this allele did not contain mutation at the enzyme active positions. The variety “Muscat blanc à Petit grain” was found to have two *VvGT7* alleles (*XB-VvGT7-4* and *XB-VvGT7-5*). *XB-VvGT7-4* was predicted to be inactive due to the mutation of enzyme active sites, whereas insertion mutation at the position 443 in *XB-VvGT7-5* may alter its enzyme activity. These varieties contained only one *VvGT14* allele with not mutation on active sites.

## Author contributions

QHP designed the experiments on vineyard samples. YW, XYL, NM, and XQ processed the samples for the analysis of volatiles and UGTs. XYL and YW provided statistical analysis and drafted the initial manuscript. QHP provided suggestions on statistical analysis and visualization of results, and revised the manuscript. All authors contributed to discussion of the results and approved the final manuscript.

### Conflict of interest statement

The authors declare that the research was conducted in the absence of any commercial or financial relationships that could be construed as a potential conflict of interest. The reviewer FH declared a shared affiliation, though no other collaboration, with several of the authors XYL, NM, XQ, and QHP to the handling Editor, who ensured that the process met the standards of a fair and objective review.

## References

[B1] Battilana J. Emanuelli F. Gambino G. Gribaudo I. Gasperi F. Boss P. K.. (2011). Functional effect of grapevine 1-deoxy-D-xylulose 5-phosphate synthase substitution K284N on Muscat flavour formation. J. Exp. Bot. 62, 5497–508. 10.1093/jxb/err231 21868399 PMC3223048

[B2] Bayrak A. Akgül A. (1994). Volatile oil composition of Turkish rose (*Rosa damascena*). J. Sci. Food Agric. 64, 441–448. 10.1002/jsfa.2740640408

[B3] Belancic A. Agosin E. Ibacache A. Bordeu E. Baumes R. Razungles A. (1997). Influence of sun exposure on the aromatic composition of Chilean Muscat grape cultivars–Moscatel de Alejandria and Moscatel rosada. Am. J. Enol. Viticult. 48, 181–186.

[B4] Bönisch F. Frotscher J. Stanitzek S. Ruhl E. Wust M. Bitz O.. (2014a). A UDP-Glucose: monoterpenol glucosyltransferase adds to the chemical diversity of the grapevine metabolome. Plant Physiol. 165, 561–581. 10.1104/pp.113.232470 24784757 PMC4044836

[B5] Bönisch F. Frotscher J. Stanitzek S. Rühl E. Wüst M. Bitz O.. (2014b). Activity-based profiling of a physiologic aglycone library reveals sugar acceptor promiscuity of family 1 UDP-glucosyltransferases from grape. Plant Physiol. 166, 23–39. 10.1104/pp.114.242578 25073706 PMC4149709

[B6] Bureau S. M. (2000). The aroma of muscat of frontignan grapes: effect of the light environment of vine or bunch on volatiles and glycoconjugates. J. Sci. Food Agric. 80, 2012–2020. 10.1002/1097-0010(200011)80:14<2012::AID-JSFA738>3.0.CO;2-X

[B7] Castellarin S. D. Matthews M. A. Di Gaspero G. Gambetta G. A. (2007). Water deficits accelerate ripening and induce changes in gene expression regulating flavonoid biosynthesis in grape berries. Planta 227, 101–112. 10.1007/s00425-007-0598-8 17694320

[B8] Dimitriadis E. Williams P. J. (1984). The development and use of a rapid analytical technique for estimation of free and potentially volatile monoterpene flavorants of grapes. Am. J. Enol. Viticult. 35, 66–71.

[B9] D'Onofrio C. Matarese F. Cuzzola A. (2017). Study of the terpene profile at harvest and during berry development of *Vitis vinifera* L. aromatic varieties *Aleatico, Brachetto, Malvasia di Candia aromatica* and *Moscato bianco*. J. Sci. Food Agric. 97, 2898–2907. 10.1002/jsfa.8126 27801497

[B10] Fenoll J. Manso A. Hellín P. Ruiz L. Flores P. (2009). Changes in the aromatic composition of the *Vitis vinifera* grape Muscat Hamburg during ripening. Food Chem. 114, 420–428. 10.1016/j.foodchem.2008.09.060

[B11] Gomez E. Martinez A. Laencina J. (2010). Changes in volatile compounds during maturation of some grape varieties. J. Sci. Food Agric. 67, 229–233. 10.1002/jsfa.2740670213

[B12] Gunata Y. Z. Bayonove C. L. Baumes R. L. Cordonnier R. E. (1985). The aroma of grapes I. Extraction and determination of free and glycosidically bound fractions of some grape aroma components. J. Chromatogr. A 331, 83–90. 10.1016/0021-9673(85)80009-1

[B13] Guth H. (1997). Quantitation and sensory studies of character impact odorants of different white wine varieties. J. Agric. Food Chem. 45, 3027–3032. 10.1021/jf970280a

[B14] Hughes J. Hughes M. A. (2009). Multiple secondary plant product UDP-glucose glucosyltransferase genes expressed in cassava (*Manihot esculenta Crantz*) cotyledons. Mitochondrial DNA 5, 41. 10.3109/10425179409039703 7894058

[B15] Lan Y. B. Qian X. Yang Z. J. Xiang X. F. Yang W. X. Liu T.. (2016). Striking changes in volatile profiles at sub-zero temperatures during over-ripening of ‘Beibinghong’ grapes in Northeastern China. Food Chem. 212, 172–182. 10.1016/j.foodchem.2016.05.143 27374521

[B16] Luan F. Mosandl A. Münch A. Wüst M. (2005). Metabolism of geraniol in grape berry mesocarp of *Vitis vinifera* L. cv. Scheurebe: demonstration of stereoselective reduction, E/Z-isomerization, oxidation and glycosylation. Phytochemistry 66, 295–303. 10.1016/j.phytochem.2004.12.017 15680986

[B17] Lücker J. Bowen P. Bohlmann J. (2004). *Vitis vinifera* terpenoid cyclases: functional identification of two sesquiterpene synthase cDNAs encoding (+)-valencene synthase and (−)-germacrene D synthase and expression of mono-and sesquiterpene synthases in grapevine flowers and berries. Phytochemistry 65, 2649–2659. 10.1016/j.phytochem.2004.08.017 15464152

[B18] Mandaokar A. Thines B. Shin B. Markus Lange B. Choi G. Koo Y. J.. (2006). Transcriptional regulators of stamen development in Arabidopsis identified by transcriptional profiling. Plant J. 46, 984–1008. 10.1111/j.1365-313X.2006.02756.x 16805732

[B19] Martin D. M. Chiang A. Lund S. T. Bohlmann J. (2012). Biosynthesis of wine aroma: transcript profiles of hydroxymethylbutenyl diphosphate reductase, geranyl diphosphate synthase, and linalool/nerolidol synthase parallel monoterpenol glycoside accumulation in Gewürztraminer grapes. Planta 236, 919–929. 10.1007/s00425-012-1704-0 22824963

[B20] Matarese F. Cuzzola A. Scalabrelli G. D'Onofrio C. (2014). Expression of terpene synthase genes associated with the formation of volatiles in different organs of *Vitis vinifera*. Phytochemistry 105, 12–24. 10.1016/j.phytochem.2014.06.007 25014656

[B21] Ong P. K. Acree T. E. (1999). Similarities in the aroma chemistry of Gewürztraminer variety wines and lychee (*Litchi chinesis Sonn*.) fruit. J. Agric. Food Chem. 47, 665–670. 10.1021/jf980452j 10563950

[B22] Osmani S. A. Bak S. Møller B. L. (2009). Substrate specificity of plant UDP-dependent glycosyltransferases predicted from crystal structures and homology modeling. Phytochemistry 70, 325. 10.1016/j.phytochem.2008.12.009 19217634

[B23] Palomo E. S. Pérez-Coello M. Díaz-Maroto M. Vi-as M. G. Cabezudo M. (2006). Contribution of free and glycosidically-bound volatile compounds to the aroma of muscat “a petit grains” wines and effect of skin contact. Food Chem. 95, 279–289. 10.1016/j.foodchem.2005.01.012

[B24] Pino J. A. Mesa J. (2006). Contribution of volatile compounds to mango (*Mangifera indica* L.) aroma. Flavour Fragr. J. 21, 207–213. 10.1002/ffj.1703

[B25] Robinson A. L. Boss P. K. Solomon P. S. Trengove R. D. Heymann H. Ebeler S. E. (2014). Origins of grape and wine aroma. part 1. chemical components and viticultural impacts. Am. J. Enol. Viticult. 65, 1–24. 10.5344/ajev.2013.12070

[B26] Schieberle P. (1991). Primary odorants of pale lager beer. Eur. Food Res. Technol. 193, 558–565. 10.1007/bf01190873

[B27] Strauss C. R. Wilson B. Gooley P. R. Williams P. J. (1986). Role of Monoterpenes in Grape and Wine Flavor. Washington, DC: ACS Symposium series-American Chemical Society.

[B28] Vilanova M. Genisheva Z. Bescansa L. Masa A. Oliveira J. M. (2009). Volatile composition of wines from cvs. Blanco lexítimo, Agudelo and Serradelo (*Vitis vin*í*fera*) grown in Betanzos (NW Spain). J. Inst. Brewing 115, 35–40. 10.1002/j.2050-0416.2009.tb00342.x

[B29] Vilanova M. Genisheva Z. Bescansa L. Masa A. Oliveira J. M. (2012). Changes in free and bound fractions of aroma compounds of four *Vitis vinifera* cultivars at the last ripening stages. Phytochemistry 74, 196–205. 10.1016/j.phytochem.2011.10.004 22071134

[B30] Wang X. (2009). Structure, mechanism and engineering of plant natural product glycosyltransferases. FEBS Lett. 583, 3303–3309. 10.1016/j.febslet.2009.09.042 19796637

[B31] Wen Y.-Q. Zhong G.-Y. Gao Y. Lan Y.-B. Duan C.-Q. Pan Q.-H. (2015). Using the combined analysis of transcripts and metabolites to propose key genes for differential terpene accumulation across two regions. BMC Plant Biol. 15:240. 10.1186/s12870-015-0631-1 26444528 PMC4595271

[B32] Wilson B. (1986). The distribution of free and glycosidically-bound monoterpenes among skin, juice, and pulp fractions of some white grape varieties. Am. J. Enol. Viticult. 37, 107–111.

[B33] Wirth J. Guo W. Baumes R. Günata Z. (2001). Volatile compounds released by enzymatic hydrolysis of glycoconjugates of leaves and grape berries from *Vitis vinifera* Muscat of Alexandria and Shiraz cultivars. J. Agric. Food Chem. 49, 2917–2923. 10.1021/jf001398l 11409987

[B34] Zhang H. Fan P. Liu C. Wu B. Li S. Liang Z. (2014). Sunlight exclusion from Muscat grape alters volatile profiles during berry development. Food Chem. 164:242. 10.1016/j.foodchem.2014.05.012 24996330

